# Influence of phthalates on glucose homeostasis and atherosclerosis in hyperlipidemic mice

**DOI:** 10.1186/s12902-015-0015-4

**Published:** 2015-04-02

**Authors:** Wei Zhou, Mei-Hua Chen, Weibin SHI

**Affiliations:** Departments of Radiology & Medical Imaging and of Biochemistry & Molecular Genetics, University of Virginia, PO Box 801339, , 266 Snyder Bldg, 480 Ray C Hunt Drive, Charlottesville, VA 22908 USA; Department of Endocrinology of Jianghuai Hospital, Huaian, Jiangsu Province 223001 China

**Keywords:** Phthalates, Type 2 diabetes, Atherosclerosis, Dyslipidemia, Mice

## Abstract

**Background:**

Phthalates are widely used as plasticizer and are considered as a typical endocrine-disrupting chemical. Epidemiological studies have associated serum or urinary phthalate metabolites with the prevalence of type 2 diabetes or related phenotypes. However, direct evidence supporting a causal role for exposure to phthalates in type 2 diabetes is lacking.

**Methods:**

To determine the potential influence of phthalates on glucose homeostasis and atherosclerosis, female apolipoprotein E-deficient (Apoe^−/−^) mice were started at 6 weeks of age on a Western diet together with or without Bis-(2-ethylhexyl) phthalate. Phthalate was administered in drinking water at a daily dosage of 100 mg/kg. We examined glucose and insulin tolerance, plasma glucose and triglyceride levels, body weight, and atherosclerotic lesions in the aortic root.

**Results:**

Two weeks after treatment, phthalate-exposed mice had significantly higher fasting blood glucose level (97.9 ± 2.1 vs. 84.3 ± 5.3 mg/dl, *P* = 0.034) and exhibited a trend of increased glucose intolerance compared to control mice. Insulin tolerance test on non-fasted mice 3 weeks after treatment revealed that phthalate had little influence on insulin sensitivity though phthalate-treated mice had a higher glucose concentration (159.2 ± 6.0 vs. 145.2 ± 3.6 mg/dl; *P* = 0.086). On the Western diet, Apoe^−/−^ mice showed a time-dependent rise in fasting plasma glucose and triglyceride levels. However, no significant differences were observed between phthalate-treated and control mice in either phenotype after 4, 8, and 12 weeks of phthalate exposure. Neither body weight nor atherosclerotic lesions of Apoe^−/−^ mice was affected.

**Conclusion:**

This study indicates that exposure to phthalates gives rise to a brief interference of glucose homeostasis but has little impact on the development of type 2 diabetes and atherosclerosis in Apoe^−/−^ mice.

## Background

Phthalates are one class of the most manufactured industrial chemicals worldwide and have been used as plasticizers to produce plastics. Due to noncovalent binding with plastics, phthalates can readily leach into foods and drinks from plastic containers. Phthalates are also present in products used for medical care and in personal cosmetics. Phthalate exposure levels for the general public are in the range of 1–10 μg/kg/day [1]. More than 75% of the population in the United States has had measurable levels of phthalate metabolites in the circulation or urine [2,3].

Recent epidemiological studies indicate that exposure to phthalates is associated with increased waist circumference, insulin resistance, and even diabetes. In cross-sectional studies, concentrations of urinary phthalate metabolites were found to be associated with increased waist circumference or insulin resistance in adult American males [4], certain age groups of females [5], self-reported diabetes among Mexican and American women [6,7], and Korean elderly [8]. Recent studies of Swedish elderly and American women show that serum or urinary levels of phthalate metabolites were associated with an increased prevalence of type 2 diabetes [9,10]. However, direct evidence supporting a causal role is scarce and even conflicting. For example, some studies showed that perinatal or adult exposure to phthalates lead to elevated blood glucose, reduced serum insulin, impaired glucose tolerance and insulin secretion in rodents [11-14] while others found the opposite results [15] or no effect at all [16]. Moreover, because the animals used in the studies did not develop type 2 diabetes, a causal link to the disease is still hypothetical.

Apolipoprotein E-deficient (Apoe^−/−^) mice is a commonly used animal model of atherosclerosis, which develops all phases of atherosclerotic lesions, progressing from the early fatty streak stage to the advanced stage with a fibrous cap and necrotic lipid core.[17] These mice also display the typical features of dyslipidemia observed in humans, including elevations in LDL cholesterol and triglyceride levels and reductions in HDL cholesterol levels.[18,19] We have found that Apoe^−/−^ mice on the C57BL/6 genetic background develop significant hyperglycemia and type 2 diabetes when fed a Western diet [20]. In this study, we examined the influences of phthalates on the development of type 2 diabetes and atherosclerosis in the Apoe^−/−^ mice.

## Methods

### Animals

Apoe^−/−^ mice, which were on the C57BL/6 genetic background, were generated from breeding pairs purchased from the Jackson Laboratory, Bar Harbor, ME. At 6 weeks of age, female mice were switched onto a Western diet containing 21% fat, 0.15% cholesterol, 34.1% sucrose, 19.5% casein, and 15% starch (TD88137, Harlan Laboratories) and maintained on the diet for 12 weeks. Female mice were chosen because they are more susceptible to atherosclerosis than male counterparts [21] and also they were used in our previous studies [22-25]. One group of mice received bis-(2-ethylhexyl) phthalate (Sigma) during Western diet consumption, and the control group received no phthalate. The phthalates were administered in drinking water at a daily dosage of 100 mg/kg of body weight for each mouse, as reported [26]. This dose has been shown to be effective in inducing estrogenic effects in mice [26]. All procedures were carried out in accordance with current National Institutes of Health guidelines and approved by the Institutional Animal Care and Use Committee.

### Glucose tolerance test (GTT) and insulin tolerance test (ITT)

GTT and ITT were performed after 2 and 3 weeks of phthalate exposure, respectively, and GTT was also performed after 11 weeks of phthalate exposure. For GTT, mice were fasted overnight and then subjected to an intraperitoneal injection of glucose (1 g/kg). Whole blood glucose was assessed with a glucometer using blood squeezed from cut tail tips immediately before and at 10, 20, 30, 60, 90, and 120 min after the injection of glucose. ITT was performed on non-fasted mice by an intraperitoneal injection of insulin (0.75 U/kg). Blood glucose was measured immediately before and at 15, 30, 45, and 60 min after insulin injection.

### Measurements of plasma glucose and triglyceride

Mice were fasted overnight before blood was collected from the retro-orbital venous plexus by inserting a micro-hematocrit capillary with the animals under isoflurane anesthesia. Ethylenediaminetetraacetic acid (EDTA) was used as an anti-coagulant. Blood samples were centrifuged for 5 min at 12,000 g at 4°C, and the resulting plasma was stored at −80°C before assay. Plasma glucose was measured with a Sigma glucose (HK) assay kit, [27] and plasma triglyceride was measured with a Thermo DMA triglyceride kit [28].

### Atherosclerotic lesion analysis

Atherosclerotic lesions in the aortic root were measured as previously reported [29,30]. Briefly, at the end of the experiments, the vasculature of mice was perfused with 4% paraformaldehyde for approximately 5 min through the heart. The aortic root and adjacent heart were excised and sectioned in 10‐μm thickness on a cryostat. Cryosections were stained with oil red O and hematoxylin and counterstained with fast green. Atherosclerotic lesion areas were measured with a Zeiss PrimoStar microscope connected with an Axiocam camera. The lesion areas of five sections with the largest readings were averaged for each mouse, and this average was used for statistical analysis.

### Statistical analysis

Values were expressed as means ± SE, with *n* indicating the number of mice. AVOVA or Student's t test were used for determining statistical significance between groups. Differences were considered statistically significant at *P* < 0.05.

## Results

### Effect on body weight

**Figure 1:**
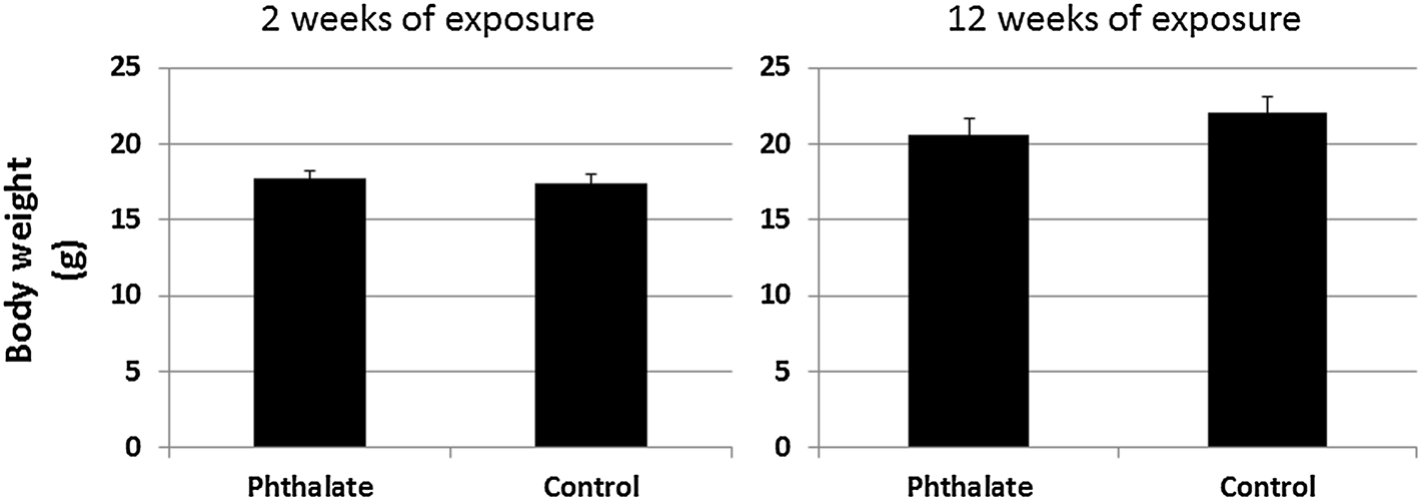
**Body weight (g) of female Apoe** ^**−/−**^ **mice at 2 and 12 weeks after the initiation of a Western diet plus phthalate.** Control mice were fed with the high fat diet but were not treated with phthalate. Phthalate was administered in drinking water at a daily dosage of 100 mg/kg of body weight. Results are means ± SE of 5 to 9 mice per group at each time point.

The body weight of Apoe^−/−^ mice was measured at 2 and 12 weeks after the initiation of phthalate exposure. As shown in Figure 1, the body weight of the mice treated with phthalate was not significantly different from that of the control mice at both the 2 week (17.7 ± 0.5 vs. 17.4 ± 0.6 g) and 12 week time points (20.6 ± 0.5 vs. 22.1 ± 0.6 g).

### Early effect on glucose homeostasis

**Figure 2:**
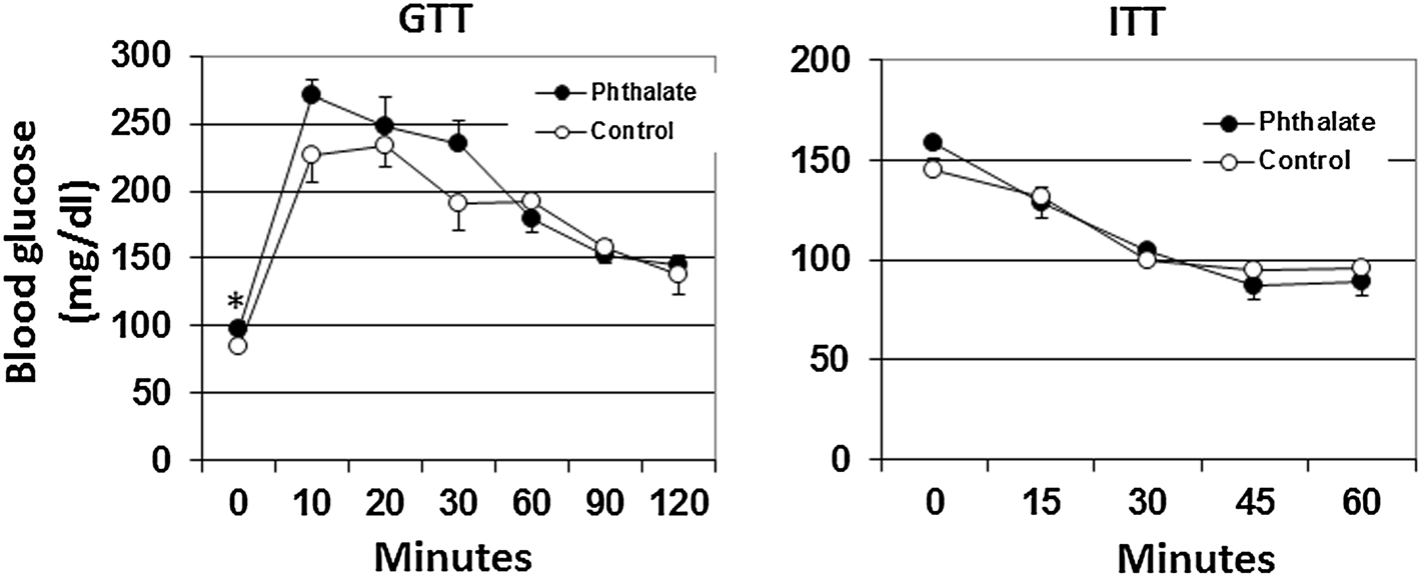
**Glucose tolerance test (GTT) and insulin tolerance test (ITT) performed on the Apoe** ^**−/−**^ **mice after being exposed to phthalate for 2 and 3 weeks, respectively.** For GTT, mice were fasted overnight and then subject to an intraperitoneal injection of glucose (1 g/kg). ITT was performed on non-fasted mice with an intraperitoneal injection of insulin (0.75 U/kg). Blood glucose concentrations were determined with a glucometer using blood taken from cut tail tips. Values are means ± SE of 5 mice. *P < 0.05 vs. control mice.

Glucose tolerance test (GTT) and insulin tolerance test (ITT) were performed on the mice that had been exposed to phthalate for 2 and 3 weeks, respectively. In response to intraperitoneally injected glucose, blood glucose levels quickly rose to the peak at the 10^th^ min for the phthalate-treated mice and at the 20^th^ min for the control mice (Figure 2). After the peak, glucose levels fell gradually. Compared with control mice, phthalate-treated mice exhibited a trend of worsened glucose intolerance: At the 10^th^ min, the phthalate group had a blood glucose level of 270.4 ± 19.6 mg/dl, as compared to a level of 226.6 ± 12.3 mg/dl in control mice (*P* = 0.10). At the 30^th^ min, phthalate-treated mice had a blood glucose level of 235.6 ± 20.4 mg/dl compared to 190.4 ± 17.2 mg/dl of control mice (*P* = 0.13). In addition, the basal fasting blood glucose level (at 0 min) was significantly higher in the phthalate-treated mice than in the control mice (97.9 ± 2.1 vs. 84.3 ± 5.3 mg/dl, *P* = 0.034). GTT was also performed after mice were exposed to phthalates for 11 weeks, but no significant differences were observed between the two groups (data not shown).

ITT was performed on non-fasted mice. In response to insulin, both group of mice showed a deep and long-lasting fall in blood glucose levels. However, the basal non-fasting blood glucose level (at 0 min) was higher in phthalate-exposed mice than in control mice (159.2 ± 6.0 vs. 145.2 ± 3.6 mg/dl), although the difference did not statistical significance (*P* = 0.086).

### Long-term effect on fasting glucose and triglyceride

**Figure 3:**
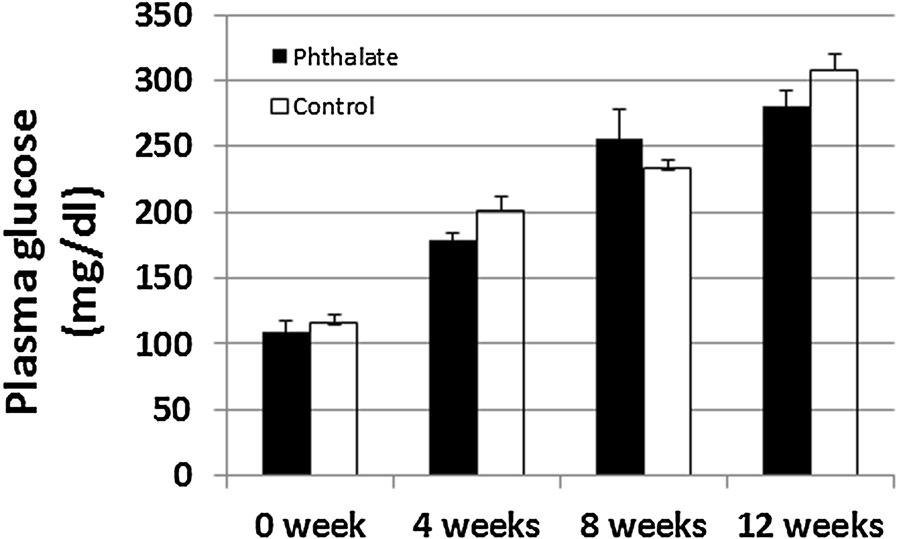
**Fasting plasma glucose levels of Apoe** ^**−/−**^ **mice before and 4, 8 and 12 weeks after the initiation of the Western diet together with or without phthalate.** Mice were fasted overnight before blood samples were collected. The X axis denotes the feeding times of phthalate and the Western diet. Results are means ± SE of 5 to 9 mice at each time point.

**Figure 4:**
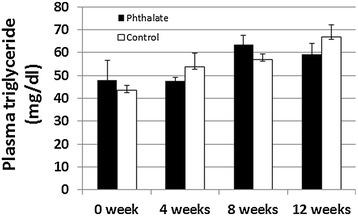
**Plasma triglyceride levels of Apoe** ^**−/−**^ **mice before and 4, 8 and 12 weeks after the initiation of the Western diet together with or without phthalate.** The same plasma samples that were used for the above glucose assessment were used for triglyceride measurements. Results are means ± SE of 5 to 9 mice at each time point.

Fasting plasma glucose and triglyceride were measured for phthalet-exposed and control mice before and after 4, 8 and 12 weeks of phthalate exposure. Both groups of mice showed a time-dependent rise in both fasting glucose (Figure 3) and triglyceride levels (Figure 4) during the 12 weeks’ feeding period on the Western diet. Fasting plasma glucose level was 109.3 ± 8.8 mg/dl in phthalate-exposed mice and 116.2 ± 6.7 mg/dl in control mice immediately before the initiation of the Western diet and rose to 279.0 ± 11.8 and 308.4 ± 12.3 mg/dl, respectively, at the end of the Western diet. Triglyceride levels rose from 48.0 ± 8.6 mg/dl in phthalate-exposed mice and 43.4 ± 2.2 mg/dl in control mice before the initiation of the Western diet to 63.6 ± 8.7 and 66.9 ± 5.2 mg/dl, respectively, by the end of the Western diet. However, no significant differences were observed between the two groups in either fasting glucose or triglyceride (*P* > 0.05).

### Effect on advanced atherosclerotic lesion formation

**Figure 5:**
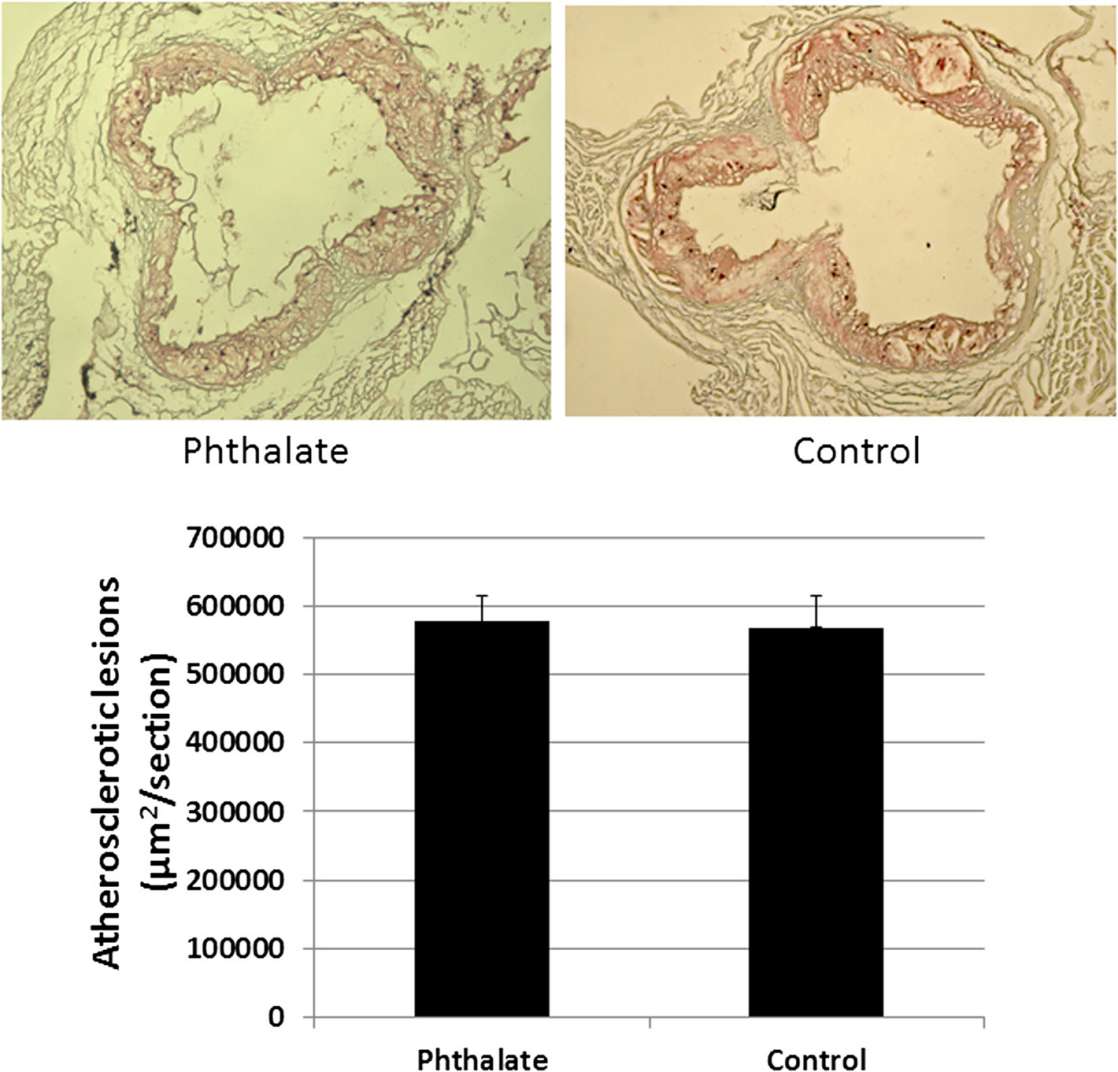
**Atherosclerotic lesions in the aortic root of Apoe** ^**−/−**^ **mice fed the Western diet together with or without phthalate.** Upper panel: Representative light microscopic pictures of aortic lesions stained with oil red O and hematoxylin. Lower panel: Quantitative measurements of atherosclerotic lesions in the aortic root stained with oil-red O. Values are mean ± SE of 8 mice for each group.

After 12 weeks of exposure to phthalate and the Western diet, Apoe^−/−^ mice were euthanized for assessment of plaque formation in the aortic root. Both groups of mice developed advanced atherosclerotic lesions that contained cholesterol clefts and necrotic areas (Figure 5). The mice exposed to phthalate had an average aortic lesion area of 577,440 ± 36,738 μm^2^/section, which was comparable to the lesion area of 568,459 ± 47,044 μm^2^/section in control mice.

## Discussion

High fat or nutrient diet is known to play an important part in the high prevalence of obesity, type 2 diabetes, and cardiovascular disease. Phthalates in plastic containers leach into foods and drinks. Thus, simultaneous intake of both high fat diet and phthalates is a daily reality. In this study, we used the Apoe^−/−^ model of atherosclerosis to evaluate the influences of phthalates on the development of diet-induced type 2 diabetes and atherosclerosis. We demonstrated that phthalates only exerted temporary effects on glucose homeostasis and glucose tolerance in the early stage of exposure (2–3 weeks) and its longer term effects on body weight, type 2 diabetes, and atherosclerosis were not significant.

We previously found that Apoe^−/−^ mice on the C57BL/6 genetic background develop significant hyperglycemia and type 2 diabetes when fed a Western diet [31,22]. Accordingly, in this study we observed a time-dependent rise of fasting plasma glucose in the Apoe^−/−^ mice on the Western diet. By the 12^th^ week, Apoe^−/−^ mice treated either with or without phthalates had a fasting glucose level surpassing 250 mg/dl (Figure 3). Diabetes is defined by fasting hyperglycemia. Fasting plasma glucose exceeding 250 mg/dl is considered diabetic for mice [32]. Clearly, these mice had developed type 2 diabetes by the 12^th^ week on the Western diet. One interesting finding in the present study was that mice treated with phthalates exhibited elevations in fasting and non-fasting glucose and impairments in glucose intolerance in the first 2 to 3 weeks of Western diet consumption, although the magnitudes of alterations were small. This finding is in agreement with previous observations from rats [11,13]. Phthalates have been shown to decrease serum insulin level [33] and interfere with insulin signal transduction in adipose tissue [13], both of which could lead to rises in blood glucose.

In this study, we found that a prolonged phthalate exposure did not affect fasting glucose of Apoe^−/−^ mice fed the Western diet. This finding agrees with a recent observation made from wild-type C57BL/6 mice fed a high fat diet [16]. The reasons for the disappearance of the effect of phthalates on glucose homeostasis over time are clear. One explanation is that as phthalates induce cytochrome P-450 and other enzymes capable of metabolizing them [34], subsequently the accelerated degradation would weaken their effects. Another explanation is that as blood glucose levels are controlled by multiple factors, the small defect caused by phthalates could be compensated by other regulators over time.

Previous studies have shown that phthalates cause a significant reduction in plasma triglyceride levels in high fat-fed wild-type C57BL/6 mice [16] and F344 rats [15]. However, we did not find any changes in plasma triglyceride level of Apoe^−/−^ mice treated with phthalates. One explanation for the discrepancy between this study and previous ones is the different dosages of phthalates: we used a much lower daily dosage (100 mg/kg) than the previous studies (500 ~ 1000 mg/kg). Another explanation is that the effect of phthalates on triglyceride might be more readily uncovered in the animals that have higher triglyceride levels (100 mg/dl in wild-type mice compared to ~50 mg/dl in the Apoe^−/−^ mice).

A recent cross-sectional study of over 1016 Caucasians aged 70 found that serum levels of a phthalate metabolite were related to carotid artery plaques [9]. However, the association was not linear rather an inverted U-shape, which did not support a causal relationship. In this study, we directly tested the effect of phthalates on plaque formation in Apoe^−/−^ mice. The present finding did not show any significant effect on plague formation as mice treated with phthalates developed atherosclerotic lesions similar to those receiving no phthalates.

Epidemiological studies have suggested a role of phthalates in obesity, but the results from the human studies was inconsistent [35,36,4,5,37,38]. In this study, we found that phthalates had no influence on body weight of Apoe^−/−^ mice on the Western diet. In a recent study, Feige et al. [16] even found that the phthalate protected wild-type C57BL/6 mice from high fat diet-induced body weight increases. We and others previously found that female B6.Apoe^−/−^ mice had no increase in body weight on the Western diet [22,31,39]. Thus, there is a possibility that female B6.Apoe^−/−^ mice are not an appropriate model for assessing the effect of phthalates on body weight.

The present study has several limitations. First, we only tested one single dosage that has been shown to be effective in altering blood glucose levels in rats [11,13]. As the magnitudes of exposure to phthalates vary greatly among the general population, a single dosage of exposure may not be able to reflect their real heath influences. Second, only female Apoe^−/−^ mice were included in the present study, while these mice do not develop obesity on a high fat diet. Third, there was a possibility that the effect of phthalates on diabetes and atherosclerosis was overwhelmed by Western diet feeding. Western diet consumption alone results in a gradual rise in fasting glucose and accelerates plaque formation; thus a small effect from phthalate might not show up.

In summary, we have observed a brief influence of phthalate at a single higher dose on blood glucose and glucose tolerance of female Apoe^−/−^ mice in the early phase of exposure. This influence disappeared within a few weeks. The overall impact of phthalate on the development of type 2 diabetes and atherosclerosis appears to be limited, although other doses that are more relevant to human situations need to be tested.
